# Diverse subpopulations of vesicles secreted by different intracellular mechanisms are present in exosome preparations obtained by differential ultracentrifugation

**DOI:** 10.3402/jev.v1i0.18397

**Published:** 2012-04-16

**Authors:** Angélique Bobrie, Marina Colombo, Sophie Krumeich, Graça Raposo, Clotilde Théry

**Affiliations:** 1 Institut Curie Centre de Recherche, Paris, France; 2 INSERM U932, Paris, France; 3 CNRS UMR144, Paris, France

**Keywords:** extracellular vesicles, exosomes, Rab proteins, markers, secretion machinery

## Abstract

Exosomes are extracellular vesicles of 50 to 100 nm in diameter, released by many cell types. Exosomes are formed inside the cell in intracellular endosomal compartments and are secreted upon fusion of these compartments with the plasma membrane. Cells also secrete other types of membrane vesicles, for instance, by outward budding from the plasma membrane, and although some of them clearly differ from exosomes by their structural features (larger size), others are possibly more difficult to separate. Here, using Rab27a inhibition to modulate exosome secretion, we show the existence of at least 2 distinct populations of vesicles after purification by classical ultracentrifugation from mouse tumor cell conditioned medium. Rab27a inhibition lead to decreased vesicular secretion of some conventional markers of exosomes (CD63, Tsg101, Alix and Hsc70) but did not affect secretion of others (CD9 and Mfge8). By electron microscopy, CD9 was observed on vesicles of various sizes, ranging from 30 nm to more than 150 nm in diameter. Flotation onto sucrose gradients showed different proportions of CD63, CD9 and Mfge8 not only in fractions of densities classically described for exosomes (around 1.15 g/ml) but also in fractions of densities over 1.20 g/ml, indicating the presence of heterogenous vesicle populations. CD9 and Mfge8 were also found in large vesicles pelleted at low speed and can thus not be considered as specific components of endosome-derived vesicles. We propose that the most commonly used protocols for exosome preparations co-purify vesicles from endosomal and other origins, possibly the plasma membrane. Future work will be required to improve techniques for accurate purification and characterization of the different populations of extracellular vesicles.

In pluricellular organisms, cells constantly exchange informations to maintain integrity of tissues and accomodate changes in their environment. Secretion of soluble molecules, which bind to receptors on the surface of other cells, has been studied since the beginning of last century. But in the last decade, communication between cells via secretion of more complex structures such as membrane vesicles has become the focus of increasing interest from the scientific community (1–3). Membrane vesicles are composed of a lipid bilayer containing transmembrane proteins and enclosing soluble proteins and RNA, and the consequences of their interaction with cells are thus much more complex than the consequences of interaction of a single ligand with a single receptor. Extracellular membrane vesicles, called “microparticles” (4), have long been known in the blood circulation, where they were considered as cell fragments or pieces of membrane shed from the cell surface (2). But another type of vesicle secretion was described in the 1980s (5,6): it involved formation of vesicles inside endosomes, followed by extracellular release of these vesicles upon fusion of the endosomes with the plasma membrane. The resulting vesicles are of similar size as the internal vesicles of endosomes, i.e. between 50 and 100 nm in diameter, and are classically purified by ultracentrifugation at 70,000 or 100,000 g (7–9). The term “exosomes” has been used since 1987 to designate these extracellular vesicles of endocytic origin (8). A similar mechanism was also proposed to result in release of vesicles called “prostasomes” in semen (10).

Our groups have started working on exosomes secreted by immune cells, in the late 1990s (9,11,12), and have shown their potential as vehicles for antigen information within the immune system. We have also initiated the first proteomic analyses of exosomes secreted by dendritic cells (12) and shown the selective presence of a subset of intracellular proteins in these vesicles. A few years ago, we have published a detailed protocol for exosome purification by differential ultracentrifugation and characterization of the resulting vesicles by a combination of approaches showing enrichment of exosomal markers (electron microscopy and Western blotting) (13): this protocol was based on the original description of methods for isolating reticulocyte exosomes by the groups of Stahl (7) and Johnstone (8), and it is often referred to in publications dealing with exosomes.

In the last 4 years, we set out to identify the molecular machinery specifically involved in exosome secretion, in order to find ways to modulate this secretion and determine the physiological functions of exosome secretion in vivo. We have thus recently demonstrated a role of the small GTPases RAB27A and RAB27B in exosome secretion by a human tumor cell line (14). In this work, secretion of exosomes was measured by the release of a set of exosomal proteins, as defined by previous work from us (9,12) and others (15): major histocompatibility complex (MHC) class II molecules, the tetraspanins CD63 and CD81, heat shock protein cognate 70 (HSC70) and the protein involved in formation of multivesicular endosomes: tumor susceptibility gene 101 (TSG101).

Here, we used the same approach of Rab27a inhibition, in order to inhibit secretion of exosomes by mouse tumor cells. In addition to the proteins analyzed in human exosomes (14), we routinely characterize mouse exosomes using CD9 and milk fat globule – epidermal growth factor – factor 8 (Mfge8), 2 markers previously identified as especially enriched in dendritic cell exosomes (12). Interestingly, this analysis allowed us to unravel a differential responsiveness to Rab27a inhibition of 3 markers, which are equally considered as specific of exosomes: 2 tetraspanins (CD63 and CD9) and a peripheral membrane-associated protein (Mfge8). Our work thus highlights the heterogeneity of the vesicle preparations commonly referred to as exosomes and shows that different structural and biochemical features are due to different molecular mechanisms of formation, and secretion.

## Results

### Selection of a shRNA sequence targeting specifically mouse Rab27a

**Fig. 1:**
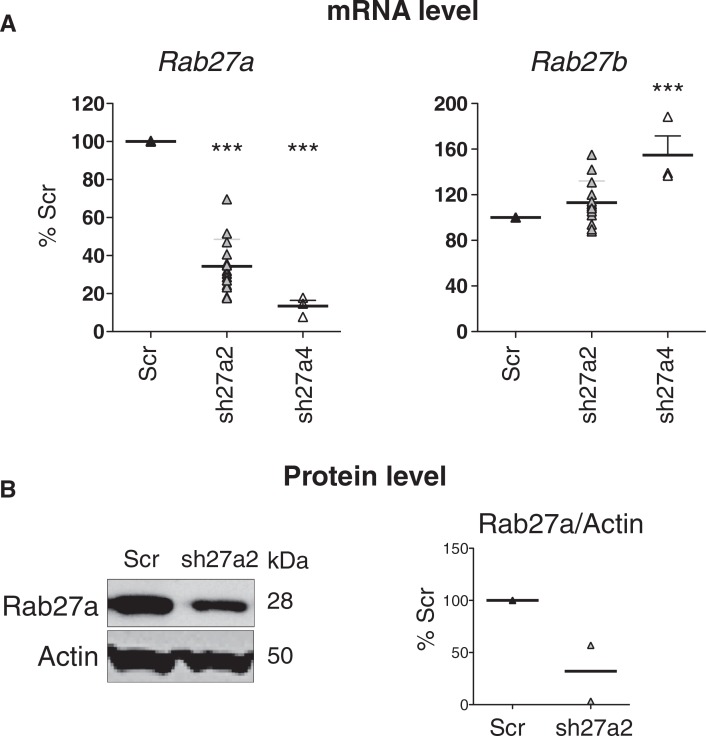
Selection of a shRNA for specific inhibition of mouse Rab27a. A, Effect of 2 different shRNA sequences specific for *Rab27a* (sh27a2 and sh27a4) on *Rab27a* and *Rab27b* expression, measured by qRT-PCR. In each experiment, *Rab27* expression in shRab27-expressing cells is compared to expression in cells expressing a control shRNA (Scr). Individual results of 3 (sh27a4) or 7 (sh27a2) experiments are shown. Both sh27a2 and sh27a4 significantly decrease *Rab27a* expression, but sh27a4 also increases *Rab27b* expression. ***p < 0.01, 1-way ANOVA, Dunnett's post-test. B, Stable expression of sh27a2 induces downregulation of Rab27a at the protein level, as shown by Western blotting on total cell lysate (left panel). Actin is shown as loading control. Individual values of arbitrary units of Rab27a/actin band intensity in 2 independent experiments is shown (right panel). In each experiment, arbitrary units obtained for Scr cells are considered as 100%.

We have shown recently that inhibition of RAB27A expression by stable transfection with shRNA in a human tumor cell line, HeLa-CIITA (a modified HeLa cell line expressing the MHC class II machinery), induces around 70% decrease in exosome secretion. This result was obtained by measuring the total amount of proteins recovered after 100,000 g ultracentrifugation, and the intensity of secretion of 4 classical markers of exosomes (CD63, TSG101, MHC class II and HSC70) (14). Here, to further extend these findings and to understand the role of exosome secretion by tumors in vivo (Bobrie, Théry et al., submitted mansucript), we analyzed the effect of Rab27a invalidation on exosome secretion by a mouse mammary carcinoma called 4T1. Following the same experimental design as in HeLa cells (14), we used lentiviruses expressing a puromycin-resistance gene and a shRNA sequence specific for *Rab27a*, or a control shRNA sequence, which does not target any mouse gene (Scr), to infect 4T1 cells. Out of 5 shRNA designed at inhibiting *Rab27a*, only 2 significantly inhibited this gene without affecting *Rab27b*, the other Rab27 isoform, in another mouse mammary carcinoma (Bobrie, Théry et al., submitted mansucript). We used these 2 shRNA in 4T1 cells and observed that one of them increased *Rab27b* expression (sh27a4, Fig. 1A). We thus used the only fully specific *Rab27a* shRNA sequence for the work described here and showed that it induced around 70% decrease of Rab27a protein in 4T1 cells (Fig. 1B).

### Inhibition of Rab27a decreases secretion of a subset of exosome markers

**Fig. 2:**
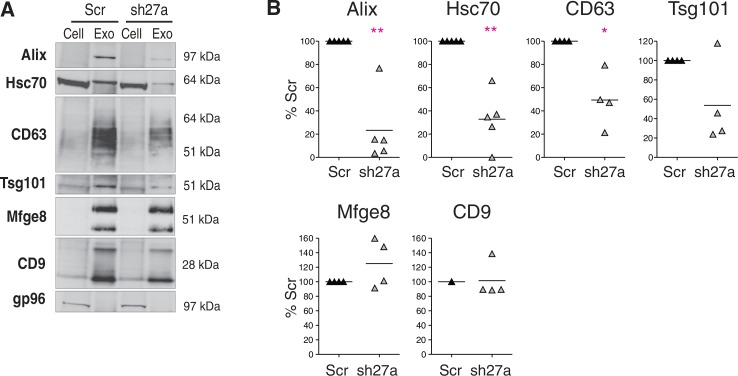
Inhibition of Rab27a decreases secretion of a subset of exosomal markers. A, Western blot characterization of exosomes (Exo) secreted by the same number of control (Scr) or shRab27a-expressing 4T1 cells (sh27a), i.e. corresponding to 2–3 µg of total proteins. 30 µg of total cell lysates (cell) were analysed in parallel. Six different exosomal markers (CD63, Alix, Tsg101, Hsc70, CD9 and Mfge8) and a negative control (gp96) are shown. B, Quantification of the amount of each marker in exosomes obtained from shRab27a-expressing cells, as compared to control cells (individual results from 5 independent experiments are shown). *p < 0.05, **p < 0.01, paired t-test.

Exosomes were purified by differential ultracentrifugation from the conditioned culture medium of Scr- or shRab27a-expressing 4T1 cells as previously described (13). The resulting 100,000 g pellet was analysed by Western blotting, using 6 different exosome markers (Fig. 2A). As expected, exosomes secreted by 4T1 cells are characterized by very strong enrichment of 2 tetraspanins (CD63 and CD9), Alix and Mfge8, as compared to cell lysates, and weaker (but clear) enrichment of 2 other classical exosome markers, Hsc70 and Tsg101. The endoplasmic reticulum-resident gp96 is used as a control for absence of cell debris in the exosome preparation. Significant decrease of secretion of 4 of these markers (CD63, Tsg101, Alix and Hsc70) was observed in shRab27a-expressing 4T1 (Fig. 2B). Surprisingly, secretion in the 100,000 g pellet of CD9 and Mfge8, 2 other markers classically used to characterize mouse dendritic or tumor cell exosomes (12,16), was not reduced in shRab27a-expressing cells (Figs. 2A and 2B).

### CD63, CD9 and Mfge8 do not behave identically after flotation of the 100,000 g pellet on sucrose gradients

**Fig. 3:**
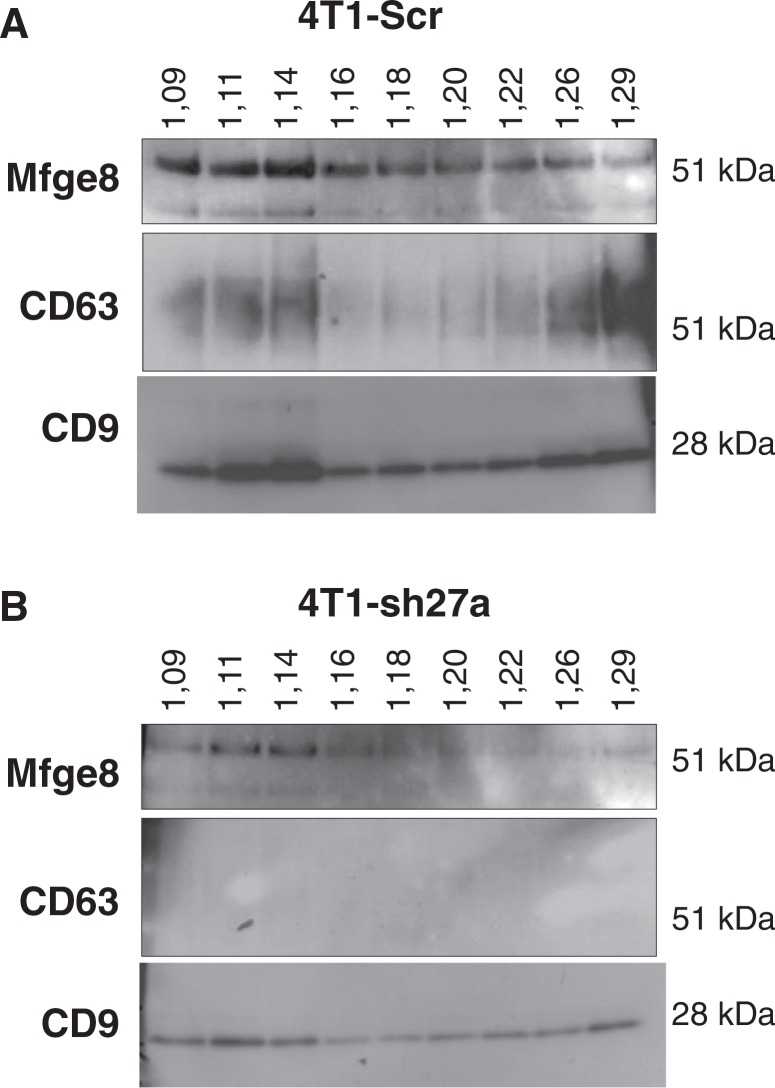
CD9, CD63 and Mfge8 display subtly different patterns after floatation on sucrose gradients. A, The 100,000 g pellet recovered from Scr-4T1 cell conditioned medium was allowed to float into a sucrose gradient. The resulting fractions were analysed by Western blot using antibodies to Mfge8, CD63 and CD9. Density of each sucrose fraction in g/ml, measured by refractometry, is indicated above the gel. Mfge8, CD9 and CD63 float at the expected sucrose density of exosomes (1.11 g/ml and mostly 1.14 g/ml). Mfge8 also floats at 1.09 g/ml, whereas a large part of the tetraspanins also floats in high density fractions (1.26–1.29 g/ml). B, Same analysis performed with 100,000 g pellets obtained from shRab27a-expressing 4T1 cells. CD9 is more abundant in the 1.11 g/ml than in the 1.14 g/ml fraction and is also abundant in the high density fractions.

Since CD9 and Mfge8 did not behave like other exosomal markers, we asked whether they could be present on different types of vesicles present in the 100,000 g pellet. A classical way to improve exosome purification after differential ultracentrifugation is to allow the pellet to float into an overlaid sucrose gradient (9,12,15,17). In these conditions, the original studies quoted above have shown that exosomal markers float at densities comprised between 1.11 and 1.19 g/ml, with slight variations of the position of the major fraction between different cell types and/or different markers. Here, we analysed the distribution of Mfge8, CD9 and CD63 among the sucrose gradient fractions, and we observed that the 3 markers did not behave identically. As shown in Fig. 3A, CD9 was present in all fractions from 1.09 to 1.29 g/ml, with a clear enrichment in the 1.11 and 1.14 g/ml fractions on one hand and, to a lower extent, in denser fractions of 1.26 and 1.29 g/ml. CD63 was also found in the same 2 sets of fractions, but it was much more abundant in the high density fractions than in the 1.11–1.14 g/ml fractions. Finally, Mfge8 was present throughout the sucrose gradient, with clear enrichment in the lightest fractions of 1.09, 1.11 and 1.14 g/ml, and no enrichment in the high density ones. When vesicles produced by shRab27a-4T1 were floated in parallel gradients (Fig. 3B), the level of CD63 was now too low to be detected, as expected from the decreased secretion level shown in Fig. 2, whereas CD9 and Mfge8 remained detectable. Interestingly, the 1.11 g/ml fraction now contained the highest amount of CD9 and as much Mfge8 as the 1.14 g/ml fraction, whereas, in vesicles from Scr-4T1, both proteins were most concentrated in the 1.14 g/ml fraction. These observations thus suggest that vesicles floating at 1.14 g/ml require Rab27a for their secretion, whereas the other fractions are not dependent on this machinery.

In addition, our results show that vesicles floating at different densities contain different relative amounts of the 3 markers. In particular, they are equally represented in the 1.14 g/ml fraction, but Mfge8 is more abundant than the 2 tetraspanins in the lowest density fraction (1.09 g/ml), whereas the high density fractions (above 1.26 g/ml) are very rich in CD63 and contain high amounts of CD9 and low amounts of Mfge8. Although such high density is normally considered to be specific of non-membranous materials like protein aggregates (which do not float into sucrose), the interpretation is different for CD9 and CD63, which are tetraspanins containing 4 hydrophobic transmembrane sequences and thereby associated to membranes. A recent article by the group of Stoorvogel has shown that, in prostasomes, the CD9-containing material found at high sucrose density after a 16-hour centrifugation (as performed here) indeed corresponds to membrane vesicles, which end up floating around 1.15 g/ml if centrifugation is performed for 62 hours (18). Our observation that CD63 is also highly enriched in these fractions thus suggest that tetraspanin-enriched vesicles display a delayed flotation capacity, as compared to vesicles containing both tetraspanins and Mfge8.

### CD9 is present on vesicles with different morphologies within the 100,000 g pellet

**Fig. 4:**
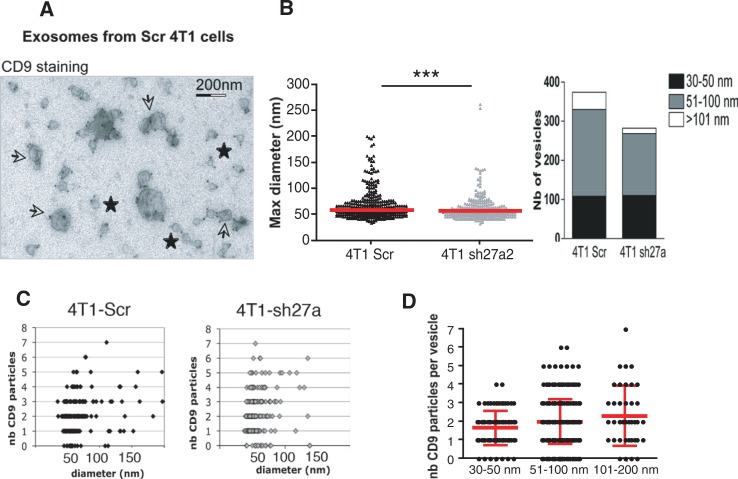
CD9 is expressed on different types of vesicles present in the 100,000 g pellet. A–C, one representative Immuno-EM analysis of exosomes secreted by control (Scr) or sh27a-4T1 cells, labelled with anti-CD9 (10 nm). A, Representative image of exosomes from 4T1-Scr. Two populations of vesicles can be observed, displaying (arrows) or not (stars) the typical cup-shaped morphology. Diameter of the latter vesicles is below 50 nm. All vesicles are positive for CD9. Scale bar: 200 nm. B, The size of each individual vesicle was measured on EM pictures. The mean size of vesicles secreted by 4T1-sh27a is decreased (left panel, ***p < 0.001), due to a decrease in the proportion of vesicles larger than 50 nm (right panel). C, Quantification of the size and number of CD9 particles on isolated vesicles obtained from Scr-4T1 (left) or sh27a-4T1 cells (right). D, Quantification of the number of CD9 particles per individual vesicles, classified according to their size (30–50 nm, 51–100 nm, 101–200 nm). Results pooled from 2 individual experiments performed on vesicles from 4T1-Scr are shown. Mean + SD is displayed.

We then used immuno-electron microscopy (immuno-EM) to determine whether morphologically different vesicle populations are present in the 100,000 g pellets (Fig. 4). Although it works well on Western Blots, the antibody directed against mouse CD63 did not perform well in immunocytochemical analysis: it labelled less than 10% of the vesicles (Supplementary Fig. S5), whereas the anti-human CD63 used in our previous study (14) labelled around 80% of human vesicles (11). The anti-Mfge8 antibody showed an even weaker staining, with some background. We could thus not conclusively determine whether all or a subtype of vesicles bear CD63 or Mfge8. By contrast, labelling with the anti-CD9 antibody allowed us to investigate the distribution of this tetraspanin among the heterogenous population of vesicles. We observed gold particles labelling CD9 not only on 50–100 nm vesicles displaying a cup-shaped morphology as previously observed for exosomes (arrows) but also on smaller non cup-shaped (30–50 nm) (stars) and on some larger cup-shaped vesicles (over 150 nm) (Fig. 4A). The size of all vesicles in 5 random fields of EM pictures (Fig. 4B) and the number of CD9 particles on 200 isolated vesicles (Fig. 4C) were then analyzed. Clustered vesicles (see several examples in Fig. 4A) were excluded from this latter analysis because it is not possible to determine to which vesicle a given CD9 particle is bound when 2 vesicles are apposed. This analysis confirmed that vesicles of all sizes contain CD9 (Fig. 4C). It also showed that the number of CD9 particles per vesicle is not higher in the largest vesicles than in the 50–100 nm vesicles (average 2 versus 2.1 gold particles /vesicle in Fig. 4D), suggesting that CD9 may be proportionally more abundant on vesicles smaller than 100 nm, than on the bigger ones. Finally, this quantification evidenced a decrease in the number of both 51–100 nm and larger vesicles in the 100,000 g pellet obtained from shRab27a-4T1 cells as compared to Scr-4T1, whereas vesicles smaller than 50 nm were as abundant in the 2 preparations (Fig. 4B). Thus, Rab27a is required for secretion of vesicles larger than 50 nm in the 100,000 g pellet and not for secretion of the smallest vesicles, which are enriched in CD9.

### Rab27a inhibition does not affect secretion of large vesicles pelleting at 10,000 g

**Fig. 5:**
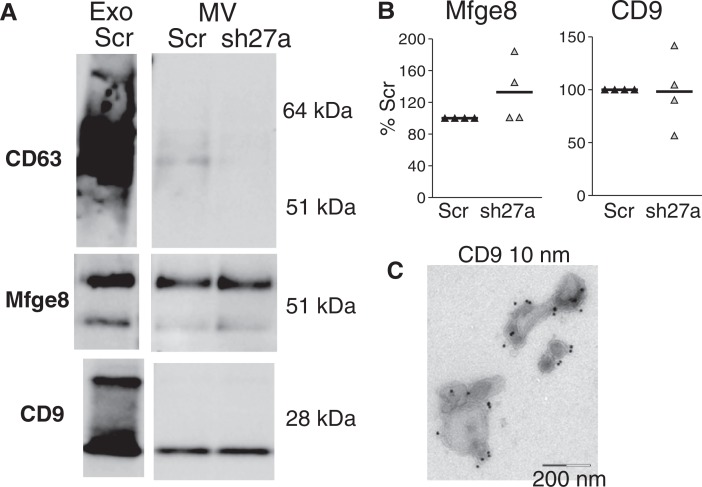
Inhibition of Rab27a does not affect secretion of CD9 and Mfge8 in vesicles pelleted at 10,000 g. A, Western blot characterization of larger vesicles pelleted at 10,000 g (MV) secreted by 16×10^6^ control (Scr) or sh27a-expressing 4T1 cells, as compared to the 100,000 g pellet (Exo) secreted by 15×10^6^ Scr cells in the same experiment. One representative Western blot showing the presence of CD9 and Mfge8 in MV, whereas CD63 is hardly detected. B, Quantification of CD9 and Mfge8 in the 10,000 g pellet obtained from sh27a-expressing cells, as compared to control (Scr) cells (individual results from 4 independent experiments). C, Immuno-EM analysis of the 10,000 g pellet after staining with anti-CD9. CD9 is present on vesicles larger than 200 nm as well as on a few exosome-sized vesicles.

Since Rab27a is involved in the secretion of a subpopulation of the vesicles recovered in the 100,000 g pellet, we asked whether it was, or not, required for secretion of large membrane vesicles pelleted at 10,000 g [usually called “microvesicles” (1,17)]. Among the markers used to characterize exosomes, we routinely detected in the vesicles pelleting at 10,000 g only CD9 and Mfge8 (Fig. 5A). CD63 was sometimes also present, but at very low level (Fig. 5A), whereas Hsc70, Tsg101 and Alix were not detectable. Even though they were both present, the relative abundance of CD9 and Mfge8 was different in exosomes than in microvesicles, with lower amounts of CD9, as compared to Mfge8, in the 10,000 g than in the 100,000 g pellet (compare Exo and MV from the same preparation in Fig. 5A). As observed for exosomes (Fig. 2B), Rab27a inhibition did not affect the level of secretion of CD9 and Mfge8 in the microvesicles (Fig. 5B), whereas it decreased the low signal observed for CD63 (Fig. 5A), suggesting that CD63 is associated with the few exosomes possibly aggregated with larger vesicles during the 10,000 g centrifugation.

We then analyzed localization of CD9 by immuno-EM on vesicles pelleted at 10,000 g. Figure 5C shows that CD9 was present on vesicles of large size (above 200 nm), as well as on some vesicles with a size consistent with that of exosomes (below 100 nm) present in the 10,000 g pellet.

**Fig. 6:**
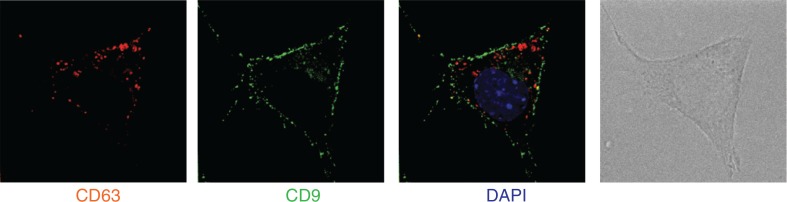
CD9 and CD63 are not present in the same intracellular locations. 4T1 cells were analysed by deconvolution microscopy after staining with anti-CD9 (green) or anti-CD63 (red). Position of the nucleus is shown by DAPI staining (blue). CD9 is mainly expressed in small patches at the cell surface, whereas CD63 accumulates in intracellular compartments.

Our observations show that Rab27a is required for secretion of vesicles bearing endosomal markers (CD63, Alix and Tsg101), proteins also described in exosomes secreted by B lymphocytes (15), dendritic cells (11) and platelets (17). Rab27a, however, is not required for secretion of smaller vesicles co-purified after differential 100,000 g ultracentrifugation, nor of larger vesicles pelleting at 10,000 g, which bear CD9 and Mfge8. In 4T1, CD63 is mainly present in intracellular compartments, whereas CD9 is found in patches at the cell membrane (Fig. 6): we thus propose that part of the CD9-bearing extracellular vesicles originate from the plasma membrane rather than from intracellular compartments. Our results also show that CD9 and Mfge8, even though they are abundant on the Rab27a-dependent exosomes, cannot be considered as specific exosome markers, since they are also present on other types of extracellular vesicles.

## Discussion

The important conclusion from our work is the direct demonstration that different types of vesicles, the secretion of which is differently dependent on Rab27a, are co-purified by the differential ultracentrifugation protocol classically used by us and others to purify exosomes (13).

Previous EM analyses of exosome preparations revealed size heterogeneity (from 30 to 100 nm) in vesicles obtained by differential ultracentrifugation of supernatants from EBV-transformed B lymphocytes (9,15), platelets (17), mouse dendritic cells (11,12) or mastocytoma and melanoma tumor cell lines (19) among others. Thus, the size of exosomes was proposed to range from 30 to 100 nm, a variability that can be explained by the fact that multivesicular endosomes in some cell types contain intraluminal vesicles of heterogenous sizes. Size of the extracellular vesicles can thus not be used as a strict feature to define exosomes. In the 4T1 cells used here, we observed that secretion of the 30–50-nm vesicles is not affected by Rab27a inhibition. Since Rab27a is known to be involved in regulated secretion from different types of intracellular compartments (20), we propose that, in the cells used here, the vesicles smaller than 50 nm do not come from intracellular compartments regulated by Rab27a. The small vesicles could thus either come from other intracellular compartments (in which case they can still be called exosomes) or directly from the plasma membrane (and we would not consider them as exosomes).

Since exosomes are smaller than the threshold of visualisation by confocal microscopy or by flow cytometry (about 200 nm), they could not be analysed reliably as single vesicles by any other methods than EM, which limited the possibilities to determine whether the differently sized vesicles were also different in terms of protein markers. Thus, it was accepted so far to characterize bulk populations of vesicles by the presence, or not, of a set of exosomal protein markers, which, if enriched in the vesicle preparations as compared to the cells secreting them, qualified vesicles as exosomes (13). But, we now show here that 2 of these exosome markers, CD9 and Mfge8, are not specific of vesicles derived from intracellular multivesicular compartments, since they are also present on larger vesicles that pellet at 10,000 g in the sequential ultracentrifugation protocol, and their secretion is not significantly affected by Rab27a inhibition as opposed to secretion of other proteins, such as CD63, Hsc70, Tsg101 and Alix (see Fig. 2).

In most cells, the bulk of CD63 is present in intracellular compartments of endosomo/lysosomal origin (21), which is also the case in the 4T1 cells used in this study (Fig. 6). CD9, by contrast, is mainly observed in 4T1 cells at the cell surface or in vesicles near the cell surface, as shown in Fig. 6. In other cells, CD9 has been described both at the cell surface and intracellularly in multivesicular endosomes (22,23). In mammary epithelial cells (24), Mfge8 not only is present throughout the cytoplasm but also binds to spots of phosphatidylserine at the cell surface, and it was shown also to associate to vesicles of different sizes, both larger and smaller than 100 nm. Thus, we propose that small vesicles budding directly from the plasma membrane, as well as vesicles formed in intracellular compartments and released by their fusion with the plasma membrane, are simultaneously secreted by the cells: ultracentrifugation at 100,000 g pellets both types of vesicles without specificity.

CD9, Mfge8 and Hsc70 had been identified in exosomes from dendritic cells in the first proteomic analysis of these vesicles that we published in 1999 (12) and Tsg101 and Alix in our following article (25). Comparative analysis, by Western blotting, of the level of these proteins in the secreting cells and their exosomes had evidenced the strong enrichment of CD9 and Mfge8 (12,26) and the clear (but weaker) enrichment of Hsc70 (12), Alix (25) and Tsg101 (27) in the exosome preparations. These observations, and the consistent identification of all these markers in numerous subsequent proteomic analyses of exosomes [analyses compiled in the Exocarta website, described initially in (16)], had led to propose these proteins as markers of exosomes. In most analyses, however, separation of the vesicles on sucrose gradients had not been performed, and the preparations contained both Rab27a-dependent and -independent vesicles. In addition, in none of these studies were exosomes and larger vesicles compared side-by-side; hence, the specificity of a given protein as a marker of exosomes, as opposed to other extracellular vesicles, was not strictly addressed. In our own previous studies, for instance, we used a portion of Mfge8 [the C1C2 domain as described in (28)] to target an antigen to secreted extracellular vesicles, and we showed that exosomes bore high amounts of the antigen (29). But the results presented here suggest that other extracellular vesicles would probably, in vivo, also bear this antigen. Thus, the increased efficiency of immune responses induced by the modified antigen in vivo could not be specifically attributed to its secretion on exosomes but more generally on extracellular vesicles. Other groups have since used the C1C2-based modification of antigens (30,31), and in these studies too, the specificity of targeting to exosomes should be re-evaluated.

Therefore, new analyses of all the proteins commonly found in exosomes should now be performed, to determine their major flotation density on sucrose gradients as compared to CD9, CD63 and Mfge8, and their enrichment on larger vesicles excluded from the exosome preparations. Such proteins include, for instance, acetylcholinesterase (AChE) or placental alkaline phosphatase (PLAP), described previously on reticulocyte (8) or tumor exosomes (32,33), but which are also present in the cell membrane (8). Density in sucrose of other proteins identified in several proteomic analyses and used randomly as markers of exosomes by different laboratories should also be determined: for instance, annexins (e.g. annexin II), and lipid raft-associated flotillins. These analyses will constitute a first step into defining the required characteristics of specific exosomal markers. Establishment of more specific purification procedures will, however, be necessary to establish a strict definition of specific exosome markers.

Indeed, none of the currently used protocols of exosome purification are likely to separate the different types of vesicles we describe here. A technique involving size-exclusion chromatography before ultracentrifugation at 100,000 g (34) allows elimination of proteins but does not separate large from small vesicles, thus *a fortiori* will not differentiate small vesicles of different densities or compositions. Several groups, including ours (14,27,35), include a filtration step over 0.22 µm filters during exosome purification: although such filtration eliminates large vesicles (as well as aggregated small vesicles), it will not separate the different vesicles of a size smaller than 220 nm. Finally, a recently commercialised method called Exoquick has been developped, aiming at fast and easy purification of exosomes. But, since the vesicles recovered bear large amounts of CD9, but low amounts of CD63, as shown on Western blots (http://www.systembio.com/microrna-research/exoquick-exosomes/technical-details), we speculate that this procedure is not specific for endosome-derived exosomes.

The use of sucrose gradients allows some separation of the different vesicles. We have observed here that, as recently described by others (18), some vesicles with high content of tetraspanins (CD9 and CD63) but low level of Mfge8 display an apparent high density in sucrose (above 1.23 g/ml). Whether these vesicles come from internal vesicles of intracellular compartments, or from portions of the plasma membrane, maybe corresponding to lipid raft-enriched subdomains, remains to be determined.

But, in addition, we observed subtle differences in the distribution of 3 membrane-associated proteins within the classical range of densities described for exosomes, i.e. 1.11 to 1.19 g/ml. Examples of slightly different densities observed for different exosomal markers from the same cellular source have been published. When analyzing simultaneously MHC class II molecules and CD81 and CD82 tetraspanins in B-EBV exosomes, Escola et al. (15) showed that the 1.13 g/ml fraction was the most enriched in MHC class II molecules, whereas CD81 was equally distributed between the 1.13 and 1.14 g/ml fractions, and CD82 between the 1.12 and 1.13 g/ml fractions. In the same line, we show here that CD9- and CD63-containing vesicles float mainly at 1.14 g/ml and to a lower extent at 1.11 g/ml, whereas Mfge8 is present equally in the same 2 fractions and a lighter one of 1.09 g/ml. In addition, we show that the CD9-containing vesicles floating at 1.14 g/ml depend on Rab27a for their secretion, whereas the vesicles floating at 1.11 g/ml are less affected by Rab27a inhibition.

Based on our results and on re-analysis of previously published work, we want to propose that sucrose gradients are not resolutive enough to separate vesicles with small differences in densities, which use different intracellular machineries for their secretion. Since they have different protein compositions, we think that the different subpopulations of extracellular vesicles, in particular, the Rab27a-dependent and -independent vesicles, should display different physiological functions. Therefore, it will be crucial in the future to design novel methods for purification and characterization of the different types of vesicles, to allow precise analysis of their respective functions.

## Materials and methods

### Cells

4T1 cells were cultured in RPMI (Life Technologies) supplemented with 10% fetal calf serum (Abcys) 2 mM glutamine, penicillin/streptomycin (100 U/mL and 100 µg/mL respectively), and 1 mM pyruvate. For stable inhibition of Rab27a expression, cells infected with shRNA-expressing lentiviruses were selected and maintained in medium containing 5 µg/mL puromycin (Life Technologies). Cells were used within 1 month after lentivirus infection. Independent experiments were performed with batches of independently infected cells.

### Reagents

Antibodies used for Western blotting: Hsc70 and gp96 (Stressgen Biotech), Tsg101 (Genetex, clone 4A10), anti-mouse CD63 (MBL, clone R5G2), Alix/AIP1 (Acris antibodies, clone 49/AIP1), anti-mouse CD9 (BD Pharmingen, clone KMC8), anti-mouse Mfge8/lactadherin (MBL, clone 18A2-G10) and actin (Millipore, clone C4). Horseradish peroxidase (HRP)-conjugated secondary antibodies were purchased from Jackson Immunoresearch except the HRP-conjugated anti-hamster antibody, which was purchased from Santa Cruz Biotechnology. Mouse anti-Rab27a was kindly provided by C. Recchi and M. Seabra (20). The same anti-CD9 and CD63 were used for immunofluorescence.

pLKO.1puro plasmids allowing expression of shRNA specific for mouse Rab27a, or a scrambled sequence of shRNA to GFP as control (Scr), and a puromycin-resistance gene (36) were kindly provided by L. F. Moita. Lentiviruses were produced as previously described and used to infect subconfluent cells in 96-well plates. Sequence of the shRNA 27a2: CCGG**CGAAACTGGATAAGCCAGCTA**CTCGAG**TAGCTGGCTTATCCAGTTTCG**TTTTTG Sequence of the shRNA 27a4: CCGG**CCAGTACACTGATGGCAAGTT**CTCGAG**AACTTGCCATCAGTGTACTGG**TTTTTG

### Quantitative RT-PCR

Total RNA were purified from 10^6^ cells using Macherey Nagel Nucleospin RNA II kit; 200 ng, as measured by Nanodrop (Thermo Scientific), was reverse transcribed with SuperScript II (Invitrogen); 1/20th of the obtained cDNA was used for quantitative PCR, performed in triplicate, in Absolute Q-PCR SYBR Green ROX Mix (Abgene) on a Lightcycler LC480 (Roche). Primers used were purchased from Qiagen (QuantiTect Primer Assay). Cycle threshold (Ct) for *Rab27a* were normalized to Ct for *Gapdh*, and results were expressed as percentage of the control shRNA-transduced cells.

### Exosome purification and characterization

Exosome purification was performed as previously described (13). Briefly, cells plated in 145 mm^2^ plates were incubated 48 hours in medium depleted from serum-derived exosomes and microvesicles by overnight ultracentrifugation at 100,000 g. Supernatants were collected and exosomes were purified by successive centrifugations: 300 g for 10 minutes; 1,000 g for 20 minutes; 10,000 g for 40 minutes and 100,000 g for 90 minutes. The 10,000 g and 100,000 g pellets were washed in large volumes of PBS and resuspended in 80 µl PBS. Producing cells were counted and proteins [obtained by lysing cells in 50 mM Tris, pH 7.5, 0.3 M NaCl, 0.5% Triton X-100, 0.1% sodium azide, with a cocktail of antiproteases (Roche)] were prepared from 4×10^6^ cells. Proteins in pellets and lysates were quantified by Micro-BCA (Thermo Scientific) in the presence of 2% SDS. Exosomes secreted by 10×10^6^ to 17×10^6^ cells (i.e around 3 µg), microvesicles (10,000 g pellet) obtained from 15×10^6^ cells or 30 µg of lysates (corresponding to around 2.10^5^ cells) were loaded on NuPAGE 4–12% BisTris gels and separated under non-reducing conditions. For Rab27a detection by Western blotting, 150 µg of lysates were analyzed in reducing conditions. Intensity of bands on Western blots was quantified using QuantityOne or ImageJ softwares.

### Electron microscopy (EM)

Immuno-EM analysis was performed as previously described (13), on pellets of purified exosomes loaded on formwar/carbon-coated grids and fixed in 2% paraformaldehyde. Grids were labelled with rat anti-mouse CD9 (KMC8, 10 µg/ml), followed by rabbit anti-rat (Dako, 1/200) and protein A-10 nm gold (UMC Utrecht, 1:60). Grids were observed at 80 kV with a CMV120 Twin Philipps Electron Microscope (PEI Company). Size of individual vesicles was measured on 5 different pictures taken at 10,000× magnification for each exosome preparation, using the iTEM software.

### Separation of vesicles on sucrose gradient

Exosomes purified from ±10^8^ cells were resuspended in 2 ml PBS-2.5M sucrose, loaded in a SW41 tube and overlaid with 15 successive 750 µl layers of PBS containing decreasing concentrations of sucrose (from 2 to 0.4 M). Tubes were centrifuged for 16 hours at 4°C at 200,000 g. One millilitre fractions were collected; sucrose density was measured on an aliquot of each on a refractometer, and fractions were diluted in 3 ml PBS, ultracentrifuged at 100,000 g for 1 hour and resuspended in PBS before analysis by SDS-page and Western blotting.

### Immunofluorescence microscopy

4T1 cells seeded on glass coverslips were fixed with 4% formaldehyde, before staining with the same anti-CD9 and anti-CD63 antibodies as for Western blots. Stainings were performed successively with anti-CD9 followed by anti-rat-Alexa488, postfixation in formaldehyde, and anti-CD63 followed by anti-rat-Alexa568. Washes were performed in PBS-0.1% BSA-0.05% Saponine and cells were stained with DAPI 1 µM before mounting. Images were acquired with a wide-field Eclipse 90i Upright Microscope (Nikon) equipped for image deconvolution. Acquisition was performed using a 100× Plan Apo VC 1.4 oil objective and a highly sensitive cooled interlined charge-coupled device (CCD) camera (Roper CoolSnap HQ2). Z-dimension positioning was accomplished by a piezoelectric motor (LVDT, Physik Instrument) and a z-dimension series of images was taken every 0.2 µm. Deconvolution was performed according to Ref. (37).
